# Supplementary material for: The dynamics of asymptomatic Plasmodium spp. infections following 10 years of malaria control interventions in Northern Sahelian Ghana
Source: PLoS Negl Trop Dis. 2026 Apr 13;20(4):e0014174. doi: 10.1371/journal.pntd.0014174 (PMC13099091; doi:10.1371/journal.pntd.0014174)
Supplement: S1 Text — (DOCX) [file pntd.0014174.s001.docx]

**S1 Text. Supporting information study design overview.**

The full details on the study site, study, population, inclusion/exclusion criteria, and data collection procedures have been previously published [1,2]. Briefly, for this study a total of 500 compounds (i.e., households) were randomly selected from each catchment area (Vea/Gowrie and Soe) in Bongo District based on the average enumeration data of 5.6 persons per compound collected in June 2012 [1]. These catchment areas were considered to be different agroecological zones (i.e., Vea/Gowrie (irrigated) vs. Soe (non-irrigated)) based on their proximity to the Vea Dam, but were otherwise similar in population size, age structure, and ethnic composition [1]. After randomly selecting an index compound in each catchment area, an equal number of male and female participants were enrolled into each of the age-stratified categories (i.e., 1–5, 6–10, 11–20, 21–39, ≥ 40 years) until the required enrolment number was reached. Pregnant women, individuals with disabilities, and individuals presenting with a serious or acute disease (including symptomatic/clinical malaria) on the day the survey was conducted were not eligible for enrollment and were excluded.

At the time the study was designed in 2012, *Plasmodium* spp. prevalence data in Bongo District were not available. Based on prevalence data between irrigated and non-irrigated areas from neighboring Kassena-Nankana Municipal District, an estimated risk ratio of 3.0 during the dry season for *Plasmodium* spp. prevalence between the catchment areas was used. Therefore, at a 95% confidence level, 80% power, and sample ratio of 1:1 between the irrigated (i.e., Vea/Gowrie) and non-irrigated (i.e., Soe) catchment areas the estimated sample size per area was 865, allowing for a 15% nonresponse rate. Based on these numbers, ~1,000 participants per catchment area (i.e., ~2,000 participants total) were recruited in all surveys. This sample size of ~2,000 participants per survey was sufficient to detect a risk ratio ≤ 0.85 for *Plasmodium* spp. prevalence between the pre-IRS (i.e., unexposed) and post-IRS or SMC (i.e., exposed) surveys with a 95% confidence level, 80% power, and a 1:1 sample ratio between the pre- and post-IRS surveys (EWS and EDS).

**REFERENCES**

1. Tiedje KE, Oduro AR, Agongo G, Anyorigiya T, Azongo D, Awine T, et al. Seasonal Variation in the Epidemiology of Asymptomatic Plasmodium falciparum Infections Across Two Catchment Areas in Bongo District, Ghana. Am J Trop Med Hyg. 2017;97: 199–212. doi:10.4269/ajtmh.16-0959

2. Tiedje KE, Oduro AR, Bangre O, Amenga-Etego L, Dadzie SK, Appawu MA, et al. Indoor residual spraying with a non-pyrethroid insecticide reduces the reservoir of Plasmodium falciparum in a high-transmission area in northern Ghana. PLOS Global Public Health. 2022;2: e0000285. doi:10.1371/journal.pgph.0000285
