# Supplementary material for: The dynamics of asymptomatic Plasmodium spp. infections following 10 years of malaria control interventions in Northern Sahelian Ghana
Source: PLoS Negl Trop Dis. 2026 Apr 13;20(4):e0014174. doi: 10.1371/journal.pntd.0014174 (PMC13099091; doi:10.1371/journal.pntd.0014174)
Supplement: S1 Table — (DOCX) [file pntd.0014174.s002.docx]

**S1 Table. Parasitological characteristics of the *Plasmodium* spp. infections (including single- and mixed-species infections) during each study time point.**

| **Parasitological parameters** | **October 2012**  (Survey 1, pre-IRS) | **October 2015** ^a^  (Survey 2, post-IRS) | **October 2017**  (Survey 3, SMC) | **November 2020**  (Survey 4, SMC) | **October 2022**  (Survey 5, SMC) |
| --- | --- | --- | --- | --- | --- |
| **Number of DBS tested** | 1,923 | 2,020 | 1,915 | 1,809 | 2,026 |
| ***P. falciparum* prevalence** (including single- and mixed-species infections) | | | | | |
| **All**  ^b^ | **1,402 (72.9)** | **788 (39.0)** | **1,214 (63.4)** | **896 (49.5)** | **1,036 (51.1)** |
| **Age groups ^c^** |  |  |  |  |  |
| < 5 years | 186 (68.6) | 66 (20.6) | 70 (23.7) | 42 (12.9) | 86 (21.9) |
| 5-10 years | 398 (82.9) | 244 (49.5) | 268 (64.3) | 152 (46.6) | 202 (45.4) |
| 11-20 years | 344 (83.3) | 251 (53.9) | 414 (84.7) | 356 (69.1) | 366 (71.3) |
| ≥ 21 years | 474 (62.5) | 227 (30.6) | 462 (64.7) | 346 (53.9) | 382 (56.6) |
| **Sex ^c^** |  |  |  |  |  |
| Female | 711 (69.0) | 389 (35.6) | 646 (62.4) | 490 (49.7) | 543 (48.5) |
| Male | 691 (77.5) | 399 (43.0) | 568 (64.6) | 406 (49.3) | 493 (54.4) |
| **Catchment area ^c^** |  |  |  |  |  |
| Soe | 801 (79.8) | 440 (43.1) | 685 (68.3) | 478 (51.1) | 528 (50.9) |
| Vea/Gowrie | 601 (65.4) | 348 (34.8) | 529 (58.0) | 418 (47.8) | 508 (51.4) |
|  |  |  |  |  |  |
| ***P. malariae* prevalence** (including single- and mixed-species infections) | | | | | |
| **All**  ^b^ | **264 (13.7)** | **29 (1.4)** | **55 (2.9)** | **133 (7.4)** | **117 (5.8)** |
| **Age groups ^c^** |  |  |  |  |  |
| < 5 years | 28 (10.3) | 0 (0.0) | 1 (0.3) | 0 (0.0) | 1 (0.3) |
| 6-10 years | 134 (27.9) | 6 (1.2) | 14 (3.4) | 21 (6.4) | 18 (4.0) |
| 11-20 years | 61 (14.8) | 13 (2.8) | 29 (5.9) | 99 (19.2) | 75 (14.6) |
| ≥ 21 years | 41 (5.4) | 10 (1.3) | 11 (1.5) | 13 (2.0) | 23 (3.4) |
| **Sex ^c^** |  |  |  |  |  |
| Female | 123 (11.9) | 13 (1.2) | 25 (2.4) | 56 (5.7) | 42 (3.8) |
| Male | 141 (15.8) | 16 (1.7) | 30 (3.4) | 77 (9.4) | 75 (8.3) |
| **Catchment area ^c^** |  |  |  |  |  |
| Soe | 166 (16.5) | 19 (1.9) | 42 (4.2) | 70 (7.5) | 50 (4.8) |
| Vea/Gowrie | 98 (10.7) | 10 (1.0) | 13 (1.4) | 63 (7.2) | 67 (6.8) |
|  |  |  |  |  |  |
| ***P. ovale* spp. prevalence** (including single- and mixed-species infections) | | | | | |
| **All** ^b^ | **110 (5.7)** | **8 (0.4)** | **76 (4.0)** | **47 (2.6)** | **26 (1.3)** |
| **Age groups ^c^** |  |  |  |  |  |
| < 5 years | 8 (3.0) | 0 (0.0) | 1 (0.3) | 0 (0.0) | 0 (0.0) |
| 6-10 years | 34 (7.1) | 1 (0.2) | 22 (5.3) | 8 (2.5) | 5 (1.1) |
| 11-20 years | 39 (9.4) | 5 (1.1) | 39 (8.0) | 28 (5.4) | 19 (3.7) |
| ≥ 21 years | 29 (3.8) | 2 (0.3) | 14 (2.0) | 11 (1.7) | 2 (0.3) |
| **Sex ^c^** |  |  |  |  |  |
| Female | 52 (5.0) | 3 (0.3) | 32 (3.1) | 18 (1.8) | 11 (1.0) |
| Male | 58 (6.5) | 5 (0.5) | 44 (5.0) | 29 (3.5) | 15 (1.7) |
| **Catchment area ^c^** |  |  |  |  |  |
| Soe | 66 (6.6) | 7 (0.7) | 57 (5.7) | 24 (2.6) | 15 (1.4) |
| Vea/Gowrie | 44 (4.8) | 1 (0.1) | 19 (2.1) | 23 (2.6) | 11 (1.1) |
|  |  |  |  |  |  |
| ^a^ For the *Plasmodium* spp. infections in October 2015, there were 2,020 participants included in the analysis: participants were excluded when no DBS was available for PCR (N = 2).  ^b^ Data reflects the number (% (n/N)) of participants sampled that were positive for *P. falciparum, P. malariae, o*r *P. ovale* spp. (including single- and mixed-species infections) as determined by *18S rRNA* PCR, relative to the total number of DBS samples tested. See Table 2 for the breakdown of the *Plasmodium* spp. groupings for single and mixed infections.  ^c^ Data reflects the number (% (n/N)) of participants sampled that were positive for *P. falciparum, P. malariae, o*r *P. ovale* spp. (including single- and mixed-species infections) as determined by *18S rRNA* PCR, relative to the total number of DBS samples tested for each age group, sex, and catchment area. See Table 1 for the demographic breakdown by age group, sex, and catchment area. | | | | | |
