# Supplementary material for: The dynamics of asymptomatic Plasmodium spp. infections following 10 years of malaria control interventions in Northern Sahelian Ghana
Source: PLoS Negl Trop Dis. 2026 Apr 13;20(4):e0014174. doi: 10.1371/journal.pntd.0014174 (PMC13099091; doi:10.1371/journal.pntd.0014174)
Supplement: S2 Table — (DOCX) [file pntd.0014174.s003.docx]

**S2 Table. Association between the study time points and *P. malariae* and *P. ovale* spp. infection prevalence using the species-specific *18S rRNA* PCR.**

| **Factor** | ***P. malariae* infection**  (including single- and mixed-species infections) **^a^** | | | | ***P. ovale* spp. infection**  (including single- and mixed-species infections) **^a^** | | | |
| --- | --- | --- | --- | --- | --- | --- | --- | --- |
|  | Unadjusted | | Adjusted ^b^ | | Unadjusted | | Adjusted ^b^ | |
|  | OR (95% CI) | *p*-value | aOR (95% CI) | *p*-value | OR (95% CI) | *p*-value | aOR (95% CI) | *p*-value |
| **Study time point (Survey, Intervention)** |  |  |  |  |  |  |  |  |
| October 2012 (Survey 1, pre-IRS) | 1.00 | - | 1.00 | - | 1.00 | - | 1.00 | - |
| October 2015 (Survey 2, post-IRS) | 0.10 (0.07-0.14) | **< 0.001** | 0.08 (0.05-0.11) | **< 0.001** | 0.07 (0.03-0.14) | **< 0.001** | 0.06 (0.03-0.13) | **< 0.001** |
| October 2017 (Survey 3, SMC) | 0.18 (0.14-0.24) | **< 0.001** | 0.14 (0.11-0.19) | **< 0.001** | 0.70 (0.52-0.94) | **0.018** | 0.62 (0.46-0.84) | **0.002** |
| November 2020 (Survey 4, SMC) | 0.50 (0.41-0.62) | **< 0.001** | 0.42 (0.34-0.53) | **< 0.001** | 0.45 (0.31-0.63) | **< 0.001** | 0.40 (0.28-0.57) | **< 0.001** |
| October 2022 (Survey 5, SMC) | 0.39 (0.31-0.48) | **< 0.001** | 0.35 (0.27-0.44) | **< 0.001** | 0.21 (0.14-0.33) | **< 0.001** | 0.20 (0.13-0.31) | **< 0.001** |
| **Age groups** |  |  |  |  |  |  |  |  |
| < 5 years | 1.00 | - | 1.00 | - | 1.00 | - | 1.00 | - |
| 5-10 years | 5.13 (3.47-7.59) | **< 0.001** | 5.14 (3.50-7.57) | **< 0.001** | 5.92 (2.94-11.9) | **< 0.001** | 5.57 (2.74-11.3) | **< 0.001** |
| 11-20 years | 6.77 (4.66-9.84) | **< 0.001** | 6.49 (4.46-9.44) | **< 0.001** | 10.1 (5.09-19.9) | **< 0.001** | 9.66 (4.84-19.3) | **< 0.001** |
| ≥ 21 years | 1.48 (0.97-2.25) | 0.067 | 1.36 (0.89-2.08) | 0.2 | 2.99 (1.48-6.04) | **0.002** | 2.81 (1.37-5.77) | **0.005** |
| **Sex** |  |  |  |  |  |  |  |  |
| Female | 1.00 | - | 1.00 | - | 1.00 | - | 1.00 | - |
| Male | 1.60 (1.33-1.92) | **< 0.001** | 1.39 (1.15-1.68) | **< 0.001** | 1.56 (1.22-1.99) | **< 0.001** | 1.41 (1.10-1.81) | **0.007** |
| **Catchment area** |  |  |  |  |  |  |  |  |
| Soe | 1.00 | - | 1.00 | - | 1.00 | - | 1.00 | - |
| Vea/Gowrie | 0.75 (0.62-0.90) | **0.002** | 0.70 (0.58-0.85) | **< 0.001** | 0.60 (0.46-0.77) | **< 0.001** | 0.57 (0.44-0.74) | **< 0.001** |
| **LLIN usage (previous night)** |  |  |  |  |  |  |  |  |
| No | 1.00 | - | 1.00 | - | 1.00 | **-** | 1.00 | **-** |
| Yes | 0.71 (0.54-0.92) | **0.011** | 1.02 (0.77-1.36) | 0.9 | 0.86 (0.56-1.30) | 0.5 | 1.08 (0.70-1.68) | 0.7 |
| **Antimalarial treatment (previous two weeks) ^c^** |  |  |  |  |  |  |  |  |
| No treatment | 1.00 | - | 1.00 | - | 1.00 | - | 1.00 | **-** |
| Treatment | 0.77 (0.63-0.94) | **0.011** | 0.55 (0.44-0.68) | **< 0.001** | 0.84 (0.62-1.12) | 0.2 | 0.76 (0.56-1.03) | 0.076 |
| OR = odds ratio; aOR = adjusted odds ratio; CI = confidence interval; LLIN = long-lasting insecticidal net  ^a^ Participants that were sick, sought treatment, but did not know if they were provided with an antimalarial treatment in the previous two weeks were excluded from the model: October 2015 (N = 79; 3.9%); October 2017 (N = 44; 2.3%); November 2020 (N = 26; 1.4%); and October 2022 (N = 6; 0.3%).  ^b^ Age groups, sex, catchment area, LLIN usage (previous night), and antimalarial treatment (previous two weeks) are adjusted for in the multivariable logistic regression model.  ^c^ Indicates those participants who reported they were sick, sought treatment, and were provided with an antimalarial treatment in the previous two weeks. For those participants who did not know if they were provided with an antimalarial treatment are coded as “Don’t know”. | | | | | | | | |
