# Supplementary material for: The dynamics of asymptomatic Plasmodium spp. infections following 10 years of malaria control interventions in Northern Sahelian Ghana
Source: PLoS Negl Trop Dis. 2026 Apr 13;20(4):e0014174. doi: 10.1371/journal.pntd.0014174 (PMC13099091; doi:10.1371/journal.pntd.0014174)
Supplement: S3 Table — (DOCX) [file pntd.0014174.s004.docx]

**S3 Table. Stratum-specific estimates for the association between age groups and *P. malariae* prevalence during each study time point.**

| **Factor** | ***P. malariae* infection** (including single- and mixed-species infections) **^a^** | | | | | | | | |
| --- | --- | --- | --- | --- | --- | --- | --- | --- | --- |
|  | **October 2012 (Survey 1, pre-IRS)** | **October 2015**  **(Survey 2, post-IRS)** | | **October 2017**  **(Survey 3, SMC)** | | **November 2020**  **(Survey 4, SMC)** | | **October 2022**  **(Survey 5, SMC)** | |
|  | aOR ^b^ | aOR (95% CI) ^b^ | *p-value* | aOR (95% CI) ^b^ | *p-value* | aOR (95% CI) ^b^ | *p-value* | aOR (95% CI) ^b^ | *p-value* |
| **Age groups** |  |  |  |  |  |  |  |  |  |
| < 5 years | 1.00 | 0.01 (0.00-0.08) | **< 0.001** | 0.04 (0.00-0.14) | **< 0.001** | 0.01 (0.00-0.07) | **< 0.001** | 0.03 (0.00-0.11) | **< 0.001** |
| 5-10 years | 1.00 | 0.03 (0.01-0.06) | **< 0.001** | 0.07 (0.04-0.13) | **< 0.001** | 0.15 (0.09-0.25) | **< 0.001** | 0.10 (0.06-0.17) | **< 0.001** |
| 11-20 years | 1.00 | 0.16 (0.08-0.29) | **< 0.001** | 0.35 (0.21-0.55) | **< 0.001** | 1.30 (0.92-1.86) | 0.14 | 0.97 (0.68-1.41) | 0.9 |
| ≥ 21 years | 1.00 | 0.24 (0.11-0.46) | **< 0.001** | 0.21 (0.10-0.42) | **<0 .001** | 0.35 (0.18-0.64) | **< 0.001** | 0.59 (0.35-1.00) | **0.048** |
| aOR= adjusted odds ratio; CI= confidence interval  ^a^ Participants that were sick, sought treatment, but did not know if they were provided with an antimalarial treatment in the previous two weeks were excluded from the model: October 2015 (N = 79; 3.9%); October 2017 (N = 44; 2.3%); November 2020 (N = 26; 1.4%); and October 2022 (N = 6; 0.3%).  ^b^ Sex, catchment area, LLIN usage (previous night), and antimalarial treatment (previous two weeks) are adjusted for in the multivariable logistic regression model. | | | | | | | | | |
