# Supplementary material for: The dynamics of asymptomatic Plasmodium spp. infections following 10 years of malaria control interventions in Northern Sahelian Ghana
Source: PLoS Negl Trop Dis. 2026 Apr 13;20(4):e0014174. doi: 10.1371/journal.pntd.0014174 (PMC13099091; doi:10.1371/journal.pntd.0014174)
Supplement: S1 Fig — Proportion of single- and mixed-species infections (A-C) in the population and (D-F) by age group for P. falciparum (green), P. malariae (blue), and P. ovale spp. (red) during each study time point. For each Plasmodium spp. darker shades represent those isolates with single-species infections, while the lighter shades denote those isolates with mixed-species infection (including double- and triple-species infections). Blank or white spaces indicate no infections detected by the species-specific 18S rRNA PCR. For the exact timing of the IRS and SMC interventions please see Fig 1. (DOCX) [file pntd.0014174.s006.docx]

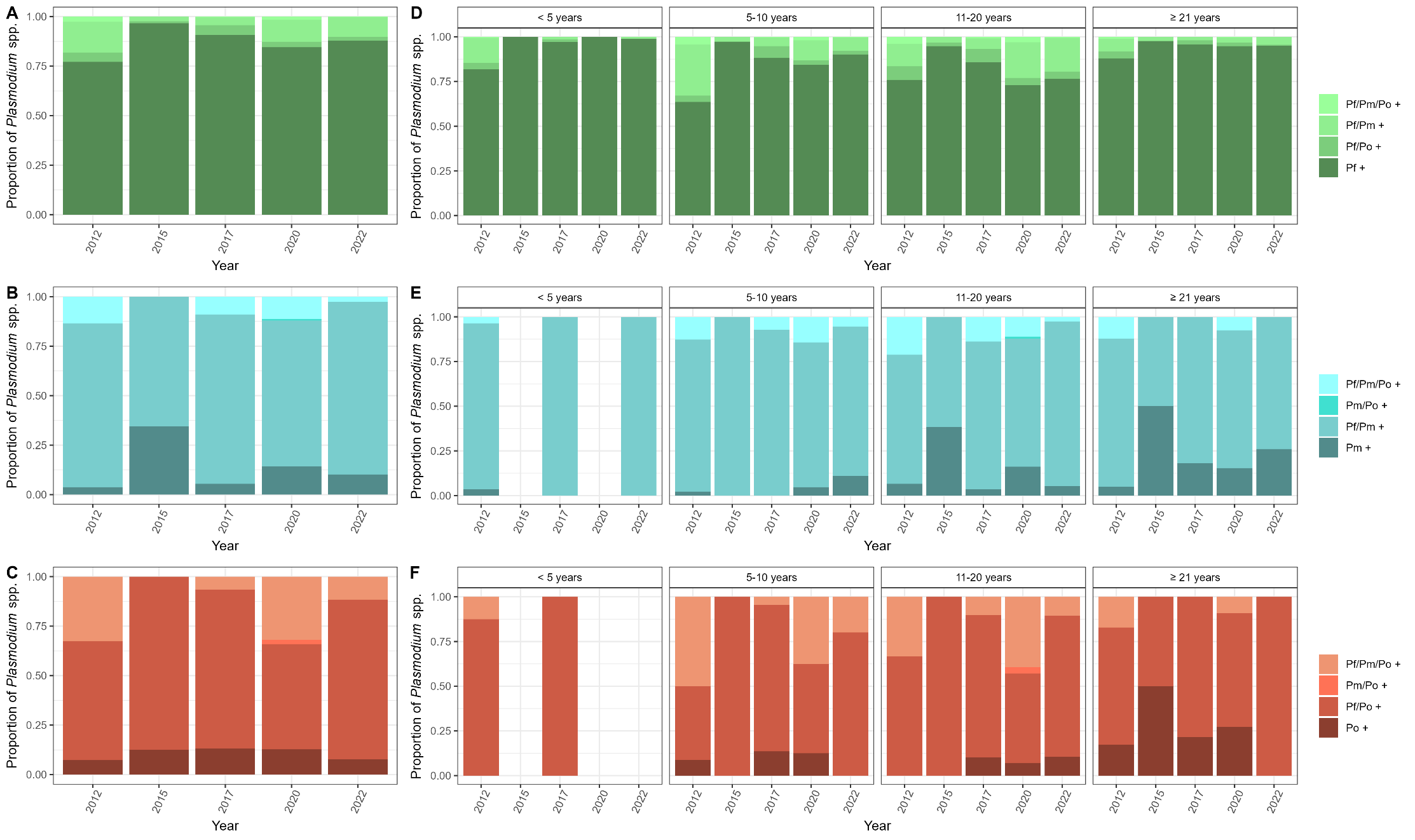


**S1 Fig.** **Proportion of single- and mixed-*Plasmodium* spp. infections identified using the species-specific *18S rRNA* PCR from October 2012 (Survey 1, pre-IRS), October 2015 (Survey 2, post-IRS), October 2017 (Survey 3, SMC), November 2020 (Survey 4, SMC), to October 2022 (Survey 5, SMC).** Proportion of single- and mixed-species infections (**A-C**) in the population and (**D-F**) by age group for *P. falciparum* (green)*, P. malariae* (blue), and *P. ovale* spp. (red) during each study time point. For each *Plasmodium* spp. darker shades represent those isolates with single-species infections, while the lighter shades denote those isolates with mixed-species infection (including double- and triple-species infections). Blank or white spaces indicate no infections detected by the species-specific *18S rRNA* PCR. For the exact timing of the IRS and SMC interventions please see Fig 1.
